# Genetic mapping and developmental timing of transmission ratio distortion in a mouse interspecific backcross

**DOI:** 10.1186/1471-2156-11-98

**Published:** 2010-11-03

**Authors:** Chevonne D Eversley, Tavia Clark, Yuying Xie, Jill Steigerwalt, Timothy A Bell, Fernando PM de Villena, David W Threadgill

**Affiliations:** 1 Department of Genetics, Curriculum in Genetics and Molecular Biology, and Carolina Center for Genome Sciences, University of North Carolina, Chapel Hill, NC 27599, USA; 2 Department of Genetics, North Carolina State University, Raleigh, NC 27695, USA

## Abstract

**Background:**

Transmission ratio distortion (TRD), defined as statistically significant deviation from expected 1:1 Mendelian ratios of allele inheritance, results in a reduction of the expected progeny of a given genotype. Since TRD is a common occurrence within interspecific crosses, a mouse interspecific backcross was used to genetically map regions showing TRD, and a developmental analysis was performed to identify the timing of allele loss.

**Results:**

Three independent events of statistically significant deviation from the expected 50:50 Mendelian inheritance ratios were observed in an interspecific backcross between the *Mus musculus *A/J and the *Mus spretus *SPRET/EiJ inbred strains. At weaning *M. musculus *alleles are preferentially inherited on Chromosome (Chr) 7, while *M. spretus *alleles are preferentially inherited on Chrs 10 and 11. Furthermore, alleles on Chr 3 modify the TRD on Chr 11. All TRD loci detected at weaning were present in Mendelian ratios at mid-gestation and at birth.

**Conclusions:**

Given that Mendelian ratios of inheritance are observed for Chr 7, 10 and 11 during development and at birth, the underlying causes for the interspecific TRD events are the differential post-natal survival of pups with specific genotypes. These results are consistent with the TRD mechanism being deviation from Mendelian inheritance rather than meiotic drive or segregation distortion.

## Background

Commonly used inbred mouse strains, which trace their genetic ancestry primarily to the *Mus musculus domesticus *subspecies [1], have extensive interspecific polymorphic differences when compared to *Mus spretus*. Because of the large number of polymorphisms that are distributed across the genome, interspecific crosses are frequently used to map genes responsible for variation in a variety of phenotypic traits [2]. In crosses between *M. musculus *and *M. spretus *only interspecific backcrosses using hybrid females are possible since hybrid males are sterile. However, interspecific backcrosses often result in skewed distributions in the inheritance of polymorphic alleles from the hybrid females, a phenomenon called transmission ratio distortion (TRD) [3-8]. Transmission ratio distortion is defined as statistically significant deviation from the expected 1:1 Mendelian ratios of allele inheritance, resulting in a reduction of the expected progeny of a given genotype.

Transmission ratio distortion involving *M. spretus *crosses was first identified during linkage testing on Chromosomes (Chrs) 2, 4 and 10 [8-11]. Subsequent efforts attempted to map the causative loci influencing TRD in four backcrosses involving *M. spretus *[6]. Transmission ratio distortion has also been observed in wild *M. musculus *populations involving Chr 1 and in commonly derived inbred strains on Chr 11 [12-15].

Among the causes of TRD are meiotic drive, segregation distortion (SD), and deviation from Mendelian inheritance (DMI) [6]. The defining characteristic of meiotic drive is that TRD occurs during female meiosis [16]. Consequently, the resulting gametes are not lost and fertility is unaffected, but the inheritance of adjacent neutral polymorphisms is affected [17,18]. Meitoic drive is one of the more common examples in which a "selfish gene" drives the preferential selection and fertilization of an oocyte [6,17]. An example of meiotic drive at the second meiotic division can be seen in the DDK syndrome at the *Om *locus on mouse Chr 11 [19,20].

Segregation distortion is due to a chromosomal transmission imbalance that typically occurs after meiosis but prior to fertilization. This mechanism is responsible for the *SD *system in *Droshophila melanogaster *and the mouse *t*-haplotype [21-25]. Finally, DMI occurs as a result of post-fertilization lethality of embryos or neonates with a particular genotype. Therefore, DMI can be used to map loci at which specific alleles have detrimental effect on survival. This is particularly interesting in crosses between closely related species because DMI may provide an important tool to study the genetics of speciation.

In this study we report three independent occurrences of TRD caused by post-meiotic lethality in a single interspecific backcross population between A/J (*M. musculus*) and SPRET/EiJ (*M. spretus) *mouse inbred strains. Preferential transmission of *M. musculus *alleles is observed on Chr 7 and of *M. spretus *alleles on Chrs 10 and 11. In addition, the Chr 11 TRD is modified by a locus on Chr 3. All three loci showing TRD are consistent with a DMI cause since allele-specific losses are not observed until after birth.

## Results

The number of progeny inheriting S or A alleles from an ASF1 female backcrossed to an A male was used to measure transmission frequencies across the mouse genome and to detect TRD. Three genomic intervals were detected that showed non-Mendelian inheritance (Table 1). Transmission ratio distortion favoring A alleles was observed on Chr 7 (χ^2 ^= 7.87; p = 0.005), while elevated frequencies of S alleles were observed on Chr 10 (χ^2 ^= 30.68; p = 3.0 × 10^-8^) and Chr 11 (χ^2 ^= 19.93; p = 8.0 × 10^-6^). There was no difference in TRD presence and level between female and male progeny (data not shown).

**Table 1:** SNP markers displaying TRD

		Observed			A allele		
Chromosome	Marker	AA	AS	Expected	Transmission	**χ**^**2**^	p-value
Chr 7	*rs8260829*	139	96	117.5	59.1%	7.87	0.005
Chr 10	*rs4228380*	73	157	115	31.7%	30.68	3.0E-08
Chr 11	*rs3707772*	82	150	116	35.3%	19.93	8.0E-06

**Figure 1:**
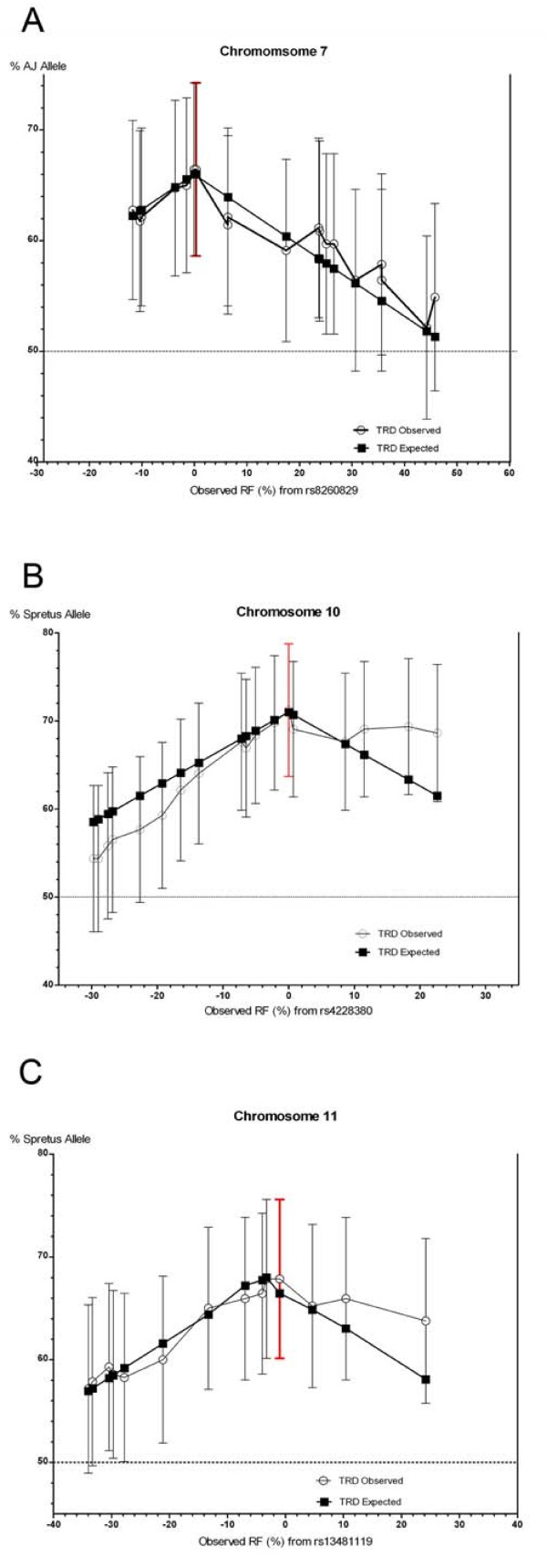
**Genetic maps of TRD**. (A) Percent transmission of A alleles when fit to a single-locus model at rs8260829 on Chr 7. (B) Percent transmission of S alleles when fit to a single-locus model at rs4228380 on Chr 10. (C) Percent transmission of S alleles when fit to a single-locus model at rs13481119 on Chr 11. Open circles represent the distortion observed. Darkened squares represent the level of distortion expected with a single distorting locus positioned at the maximally distorted marker on each chromosome. Red lines indicate maximal likely location of the TRD causing alleles. The observed recombination distances are indicated on the x-axis and only markers showing distortion are represented.

An approach developed to map TRD to a single locus or multiple linked loci was used to identify the location of the distorted loci with the minimum goodness-of-fit (GF) for each TRD region [6,19]. The distribution of allele frequencies along Chr 7 can be explained by TRD at a single locus located within a 6 cM interval centered at 27.8 cM (Figure 1A). The best GF location was determined by incrementally shifting 0.2 cM away from *rs8260829 *at 28 cM (GF = 10.003, 31 d.f., not significant p = 0.99).

Among 19 SNPs on Chr 10, a single peak was evident at *rs4228380 *(Figure 1B). The model posits that a single distorted locus is located at 48.5 cM with an expected distortion of 71%. This is in good agreement with the predictions of the GF model (GF = 12.08, 19 d.f., not significant p = 0.88). Incremental adjustments of the location and TRD had no affect on the minimum GF.

Chromosome 11 shows broad distortion spanning 16 SNPs incrementally spaced with a maximum peak of 67% TRD (Figure 1C). After adjusting the location of GF and TRD, the best GF was found to be near *rs13481119 *at 45 cM distal to the centromere (GF = 3.631, 16 d.f., not significant p = 0.99).

In addition to reduced fitness of gametes inheriting the A allele on Chr 11, transmission of alleles on Chr 11 was strongly modified by co-segregating alleles on Chr 3 (Table 2). Gametes inheriting A alleles on both Chr 3 and 11 showed significantly reduced fitness. The strongest interaction occurred between a locus at approximately 4.6 cM distal to the centromere on Chr 3 and 46.0 cM distal to the centromere on Chr 11 (χ^2 ^= 11.89, p = 0.0005). No interactions with other regions of the genome were detected for TRD on Chrs 7 or 10.

**Table 2:** SNP markers showing an interaction influencing TRD

		Chr 3 *rs3694133*		**χ**^**2**^	p-value
		AA	AS		
**Chr 11** ***rs13481119***	**AA** **AS**	15 60	30 33	11.89	0.0005

Post-weaning mortality was minimal and could not account for the deficit of specific alleles in the TRD intervals, indicating that allele loss leading to TRD occurs before weaning. To determine the timing of allele loss, (ASF1)A neonates and embryos were produced and TRD intervals genotyped using informative microsatellite markers. Mendelian ratios were observed in embryos and in neonates for each of the three TRD intervals (Table 3). These data show that the TRD is not a result of a meiotic selection in the ASF1 heterozygous dam or a preferential survival of embryos. In addition, there was no correlation between placenta or birth weight and genotype (data not shown). Although genotyping was not performed on adult mice, these experiments were conducted using inbred mouse strains at the same facility under the same conditions therefore we expect DMI to be present and reproducible. Consequently, the TRD for loci on Chrs 7, 10 and 11 in the interspecific backcross is caused by DMI that occurs post-natally between birth and weaning.

**Table 3:** Timing of the TRD effect

Marker	Observed		Expected	**χ**^**2**^	p-value
	AS	AA			
**Pre-natal**					
*D7Mit309*	28	31	29.5	0.15	0.70
*D10Mit108*	30	30	30	0.00	1.00
*D10Mit145*	28	33	30.5	0.41	0.52
*D11Mit152*	27	31	29	0.28	0.60
*D11Mit225*	31	28	29.5	0.15	0.70
*D11Mit338*	35	25	30	1.67	0.20
**Post-natal**					
*D7Mit309*	42	44	43	0.05	0.83
*D10Mit108*	47	37	42	1.19	0.28
*D10Mit145*	46	41	43.5	0.29	0.59
*D11Mit152*	38	48	43.5	1.39	0.24
*D11Mit225*	42	46	44	0.18	0.67
*D11Mit338*	40	43	43	0.42	0.52

## Discussion

Three independent loci causing strong transmission ratio distortion favoring A alleles on Chr 7 and S alleles on Chrs 10 and 11 were identified. This is the third report of TRD involving Chr 11, but the first to document an interaction with Chr 3 [12,14]. The distorting losses all occur after birth but prior to weaning and are likely the result of allelic incompatibilities influencing pup survival.

The region of maximum distortion on Chr 7 has been reported to harbor many imprinted genes influencing fetal and placental growth, postnatal growth, lethality and viability [26]. Most notably, this region contains orthologs of the imprinted gene cluster involved in the neurological disorders Prader-Willi Syndrome and Angelman Syndrome; Prader-Willi Syndrome is caused by the lack of a functional paternal copy (maternally imprinted), while Angelman Syndrome is caused by a lack of maternal copy [27]. Investigation into the genes underlying Prader-Willi Syndrome reveals that *Necdin *mutants display respiratory instability and die within the first week after birth [28]. Forty-five percent of mice with maternally transmitted Angelman Syndrome die seven days after birth or display a reduction in post-natal growth and viability [29,30].

It is more plausible that the distortion evident in this study was due to maternally inherited chromosomes with a paternal imprint. Angelman Syndrome, rather than Prader-Willi Syndrome, is caused by a loss of maternal genetic contribution. Although, Prader-Willi Syndrome causes postnatal lethality, the phenotype of Angelman Syndrome is not 100% penetrant and is consistent with the abundance of homozygous animals in the interspecific backcross. The TRD on Chr 7 could be due to variants within the orthologous Angelman Syndrome imprinting center or strain-specific methylation patterns causing silencing of maternally transmitted S allele(s). Meiotic transmission of the A allele by the heterozygous mother results in a viable homozygous mouse and is unlikely to be influenced by methylated silencing. Imprinting defects have been reported in interspecific crosses [31-33], and disregulation of imprinting is thought to be common cause of hybrid disgenesis [34].

The TRD on Chr 10 has been previously reported using C57BL/6J and SPRET/EiJ backcrosses [7,10]; the genetic similarity between A/J and C57BL/6J suggests that the distorted locus is conserved in these strains [35]. In previous reports the Chr 10 TRD was reported to extend from *Myb *to *Ifg1 *with an apex at *Col6a1/Col6a*, near *D10Mit242 *(41.2 cM) [10]. A similar TRD spanning more than 15 cM and with maximum peak at *D10Bir9 *(30 cM) was also reported for an interspecific backcross [7]. Reciprocal crosses confirmed a preferential selection of heterozygous alleles over homozygous C57BL/6J alleles similar to the data presented here.

Unlike Chr 7, the TRD interval on Chr 10 is not known to contain imprinted genes. It is possible that the TRD on Chr 10 may be influenced by global strain-specific methylation differences resulting in an incompatibility that is not completely penetrant.

Although epistatic interactions influencing TRD have been reported [6,36,37], the current data reports an independent modifier that is not located in a region of statistically significant distortion yet has a striking effect on the Chr 11 TRD. Transmission ratio distortion on Chr 11 is increased among backcross progeny homozygous for A/J genotypes on Chr 3 suggesting a maladaptive incompatibility between *Chr 3^AA ^*and *Chr 11^AA^*. There is a possibility for a third undetected modifier influencing the TRD of Chr 11 since the least represented progeny are *Chr 3^AA^*, *Chr 11^AA^*.

Although there are now two reports of TRD occurring in a similar region on Chr 11, the data suggests different mechanism are responsible. Unlike the previous report, which reported a sex skewed TRD without a Chr 3 interaction in a cross between two *M. musculus*-derived strains [12], the TRD of Chr 11 reported here does not differentially affect the sexes. Additionally, it is unlikely to be due to the early embryonic lethal DDK syndrome since the timing of TRD is between birth and weaning. Further phenotypic characterization of developing pups will be required to identify the physiological cause of DMI on Chrs 7, 10 and 11 in mouse interspecific backcrosses.

## Conclusion

In summary, Mendelian ratios of inheritance occur for Chr 7, 10 and 11 during development and at birth, but not at weaning. Additionally, Chr 3 genotypes influence TRD of Chr 11. The data indicate a differential post-natal survival of pups with specific genotypes. These results are consistent with the TRD mechanism being deviation from Mendelian inheritance rather than meiotic drive or segregation distortion.

## Methods

### Genetic crosses

All mice were obtained from The Jackson Laboratory (Bar Harbor, ME). Female A/J (A) mice (*M. musculus) *were crossed to male SPRET/EiJ (S) mice (*M. spretus*) to generate interspecific hybrids. In all crosses dams are listed first and sire second. Female ASF1 hybrids were backcrossed to A/J males to generate a segregating population of 235 (ASF1)A backcross mice in the initial experiment. Mice were euthanized at eight months of age and DNA extracted from tail and liver tissue using a Purgene DNA Extraction kit (Promega, Madison, WI).

Additional cohorts of (ASF1)A offspring were generated and euthanized within two days of birth (*n *= 88), or pre-natally between 12.5 - 19.5 days post-coitus (*n *= 60). For pre-natal samples, placenta and embryo weights were recorded and DNA was extracted from tail tissue by phenol-chloroform extraction.

All animal studies were approved by the Institutional Animal Care and Use Committee of University of North Carolina at Chapel Hill.

### Genotyping

DNA samples from (ASF1)A mice were commercially genotyped using 254 informative single nucleotide polymorphisms (SNP) markers (Illumina, San Diego, CA). Additional genotyping was done using a custom Sequenom MassArray SNP Genotyping platform containing 182 SNP markers (Geneseek, Lincoln, NE). Sequenom SNP markers were selected from NCBI Build 37 placed at approximately 10-15 cM intervals (Additional file 1, Table S1).

The microsatellite markers *D7Mit309, D10Mit145, D10Mit108, D11Mit338, D11Mit152 *and *D11Mit225 *(Operon Biotechnologies, Huntsville, AL) were used to test for TRD in embryos and neonates. Standard PCR methods were used [38,39]. PCR products were fractionated using 2%-4% agarose gels and stained with ethidium bromide for visualization.

### Statistical analysis

Evaluation of loci for TRD was performed on each cross using Chi square analysis with one degree of freedom. A corrected version of Montagutelli's GF test for single or multiple loci showing TRD was used as previously described [6,19]. A correlation analysis was performed to detect associations between genotype and placenta or birth weight. Pair-wise analyses were also performed genome-wide with the primary TRD loci to detect modifier loci. Significance thresholds were corrected for multiple testing [40].

## Authors' contributions

Conceived and designed the experiments: CDE and DWT. Performed the experiments: CDE, TC, YX, JS and TAB. Analysed the data: CDE, YX, FPMV and DWT. Wrote the paper: CDE, FPMV and DWT. All authors read and approved the final manuscript.

## Supplementary Material

Additional file 1 **Markers used in genetic mapping**. Table listing of the SNP markers used in the genetic analysis. Click here for file
