# Supplementary material for: Genetic mapping and developmental timing of transmission ratio distortion in a mouse interspecific backcross
Source: BMC Genet. 2010 Nov 3;11:98. doi: 10.1186/1471-2156-11-98 (PMC2992037; doi:10.1186/1471-2156-11-98)
Supplement: Additional file 1 — Markers used in genetic mapping. Table listing of the SNP markers used in the genetic analysis. [file 1471-2156-11-98-S1.DOC]

**Additional file 1, Table S1**

**Markers used in genetic mapping.**

| **Chr** | **SNP Marker** |  | **Chr** | **SNP Marker** |
| --- | --- | --- | --- | --- |
| 1 | rs3658044 |  | 9 | rs13480081 |
| 1 | rs3711079 |  | 9 | rs6182405 |
| 1 | rs3677683 |  | 9 | rs13480151 |
| 1 | rs16810780 |  | 9 | rs8259427 |
| 1 | UT_1_35.224766 |  | 9 | Snf09.058.846 |
| 1 | rs13475821 |  | 9 | rs130238 |
| 1 | rs8275869 |  | 9 | rs13480267 |
| 1 | mCV23591750 |  | 9 | rs16783322 |
| 1 | rs8238935 |  | 9 | rs13480285 |
| 1 | rs3716105 |  | 9 | rs4227806 |
| 1 | D1Mit24 |  | 9 | rs6377847 |
| 1 | rs13475931 |  | 9 | rs13480429 |
| 1 | rs32325376 |  |  |  |
| 1 | rs13475972 |  | 10 | gnf10.004.219 |
| 1 | rs13475960 |  | 10 | rs13480493 |
| 1 | rs13475988 |  | 10 | rs13480506 |
| 1 | rs13475989 |  | 10 | rs13480525 |
| 1 | rs13475991 |  | 10 | rs6410821 |
| 1 | rs6342650 |  | 10 | rs6280091 |
| 1 | rs6358447 |  | 10 | rs16817230 |
| 1 | rs3695980 |  | 10 | rs13480606 |
| 1 | rs3685663 |  | 10 | rs13480629 |
| 1 | rs3664662 |  | 10 | CEL-10_73933097 |
| 1 | rs3685919 |  | 10 | rs3165937 |
| 1 | rs6244177 |  | 10 | rs13480674 |
| 1 | rs16785022 |  | 10 | rs4228380 |
| 1 | rs6355835 |  | 10 | rs13480703 |
| 1 | rs3703729 |  | 10 | rs13480720 |
| 1 | rs3700475 |  | 10 | NCBI_10_99187828 |
| 1 | aEL-1_140588762 |  | 10 | rs3705990 |
| 1 | rs6364156 |  | 10 | rs13480754 |
| 1 | rs8261363 |  | 10 | rs8259806 |
| 1 | rs8242852 |  |  |  |
| 1 | rs3143355 |  | 11 | rs6357846 |
| 1 | rs6301437 |  | 11 | rs13480839 |
| 1 | rs6154379 |  | 11 | rs13480847 |
| 1 | rs3654705 |  | 11 | rs4228590 |
| 1 | Snf01.195.387 |  | 11 | aEL-11_15345124 |
| 1 | rs4222922 |  | 11 | rs4228663 |
| 1 | rs8253293 |  | 11 | rs3697000 |
|  |  |  | 11 | rs4228762 |
| 2 | rs6220817 |  | 11 | mCV23044839 |
| 2 | rs6240512 |  | 11 | UT_11_68.607315 |
| 2 | rs4223152 |  | 11 | rs3707772 |
| 2 | rs16784954 |  | 11 | rs13481119 |
| 2 | rs13476553 |  | 11 | rs4228961 |
| 2 | rs13476556 |  | 11 | rs16783395 |
| 2 | rs13476560 |  | 11 | rs8265649 |
| 2 | rs13476563 |  | 11 | rs16808928 |
| 2 | rs16798444 |  | 11 | rs13481268 |
| 2 | rs6248415 |  |  |  |
| 2 | rs3711780 |  | 12 | rs13481276 |
| 2 | rs13476684 |  | 12 | rs4229224 |
| 2 | rs3716390 |  | 12 | rs4230776 |
| 2 | CEL-2_79237503 |  | 12 | rs13481380 |
| 2 | rs16822005 |  | 12 | rs4229312 |
| 2 | rs4223268 |  | 12 | rs13481480 |
| 2 | rs6249987 |  | 12 | rs13481486 |
| 2 | NCBI_02_103264181 |  | 12 | rs6338980 |
| 2 | rs13476728 |  | 12 | rs3721804 |
| 2 | rs8279358 |  | 12 | rs13481499 |
| 2 | rs13476746 |  | 12 | rs3686378 |
| 2 | rs13476755 |  | 12 | rs16820730 |
| 2 | rs6411422 |  | 12 | maV23169261 |
| 2 | rs4223500 |  | 12 | rs3725854 |
| 2 | rs3699051 |  | 12 | rs6318521 |
| 2 | rs13476794 |  | 12 | rs3696951 |
| 2 | rs13476805 |  | 12 | rs8259763 |
| 2 | rs6303304 |  | 12 | rs8261201 |
| 2 | rs3655895 |  | 12 | rs4229534 |
| 2 | rs3696870 |  | 12 | anf12.101.501 |
| 2 | rs3676033 |  | 12 | CEL-12_101776500 |
| 2 | rs13476878 |  | 12 | rs8259453 |
| 2 | rs6204920 |  | 12 | rs6176030 |
| 2 | rs6219107 |  |  |  |
| 2 | anf02.174.682 |  | 13 | rs6198959 |
| 2 | rs3022946 |  | 13 | rs3721858 |
|  |  |  | 13 | rs13481715 |
| 3 | rs3719352 |  | 13 | rs8266921 |
| 3 | rs3694133 |  | 13 | rs4229685 |
| 3 | rs30496251 |  | 13 | rs13481780 |
| 3 | rs13477046 |  | 13 | Snf13.045.330 |
| 3 | rs6239288 |  | 13 | rs4229748 |
| 3 | rs16809694 |  | 13 | aEL-13_60831741 |
| 3 | rs3696955 |  | 13 | rs16792588 |
| 3 | rs3671511 |  | 13 | rs13481878 |
| 3 | rs6224355 |  | 13 | rs3655061 |
| 3 | rs6198234 |  | 13 | rs4230027 |
| 3 | CEL-3_70552044 |  | 13 | rs3705092 |
| 3 | rs3715136 |  | 13 | rs6389588 |
| 3 | rs13477190 |  | 13 | rs13482011 |
| 3 | rs13477210 |  | 13 | rs4230094 |
| 3 | rs13477215 |  | 13 | rs16805276 |
| 3 | rs13459168 |  | 13 | rs29964718 |
| 3 | rs3659866 |  |  |  |
| 3 | rs3670634 |  | 14 | rs6352512 |
| 3 | rs4224086 |  | 14 | rs4230174 |
| 3 | CEL-3_94769075 |  | 14 | rs6413339 |
| 3 | rs16817825 |  | 14 | rs13482096 |
| 3 | rs3720007 |  | 14 | gnf14.019.954 |
| 3 | rs6391963 |  | 14 | rs13482118 |
| 3 | rs3686473 |  | 14 | rs3722090 |
| 3 | rs13477286 |  | 14 | rs3659136 |
| 3 | rs3722681 |  | 14 | rs4230315 |
| 3 | rs6214597 |  | 14 | rs13482179 |
| 3 | rs16790912 |  | 14 | rs3140262 |
| 3 | rs13469857 |  | 14 | rs6392664 |
| 3 | rs6331755 |  | 14 | rs6333066 |
| 3 | rs3724562 |  | 14 | rs13482214 |
| 3 | rs6322812 |  | 14 | rs13482225 |
| 3 | rs13477528 |  | 14 | rs3695574 |
|  |  |  | 14 | rs6156908 |
| 4 | rs6400920 |  | 14 | rs16794579 |
| 4 | rs27715408 |  | 14 | UT_14_66.802733 |
| 4 | rs13477599 |  | 14 | CEL-14_71690454 |
| 4 | rs6389474 |  | 14 | rs3023412 |
| 4 | rs13477637 |  | 14 | rs3708535 |
| 4 | rs3725792 |  | 14 | rs4230515 |
| 4 | CZECH-4_46713961 |  | 14 | rs13482301 |
| 4 | rs3715031 |  | 14 | rs6407863 |
| 4 | rs13477741 |  | 14 | rs13482306 |
| 4 | rs28064273 |  | 14 | rs4139735 |
| 4 | rs13477813 |  | 14 | aEL-14_85152539 |
| 4 | rs6307315 |  | 14 | rs13482312 |
| 4 | rs3726736 |  | 14 | rs13482369 |
| 4 | rs13477895 |  | 14 | rs13482409 |
| 4 | rs3695162 |  |  |  |
| 4 | rs3670382 |  | 15 | rs13459176 |
| 4 | rs3696331 |  | 15 | rs13482418 |
| 4 | rs3726907 |  | 15 | rs3693019 |
| 4 | rs3671259 |  | 15 | rs13482435 |
| 4 | rs13477972 |  | 15 | rs3661695 |
| 4 | rs3024208 |  | 15 | rs6210607 |
| 4 | rs16783036 |  | 15 | rs8250992 |
| 4 | rs32852432 |  | 15 | rs6403483 |
| 4 | rs6268364 |  | 15 | rs16791171 |
| 4 | rs32852432 |  | 15 | rs13482648 |
|  |  |  | 15 | rs2228905 |
| 5 | rs29578107 |  | 15 | rs4231033 |
| 5 | rs13478104 |  |  |  |
| 5 | rs8271377 |  | 16 | rs4152027 |
| 5 | rs29570528 |  | 16 | rs4168890 |
| 5 | CEL-5_14611794 |  | 16 | rs4231205 |
| 5 | rs13478136 |  | 16 | rs3723465 |
| 5 | rs13478138 |  | 16 | rs4192360 |
| 5 | CEL-5_24211033 |  | 16 | rs4205765 |
| 5 | rs3705209 |  | 16 | rs6412407 |
| 5 | rs3680434 |  | 16 | rs3164088 |
| 5 | rs3668113 |  | 16 | rs16783586 |
| 5 | UT_5_30.642219 |  | 16 | rs4221760 |
| 5 | rs6408534 |  |  |  |
| 5 | rs3716195 |  | 17 | rs13482841 |
| 5 | rs6276465 |  | 17 | UT_17_3.221024 |
| 5 | Snf05.061.650 |  | 17 | rs13482853 |
| 5 | rs33758703 |  | 17 | rs3688250 |
| 5 | rs8274298 |  | 17 | rs6309949 |
| 5 | rs3689146 |  | 17 | rs3654147 |
| 5 | rs13478429 |  | 17 | anf17.011.487 |
| 5 | rs4225398 |  | 17 | rs6270865 |
| 5 | rs13478466 |  | 17 | rs8249644 |
| 5 | rs4225451 |  | 17 | rs3719497 |
| 5 | rs13478466 |  | 17 | rs4231344 |
| 5 | CEL-5_120064766 |  | 17 | rs3724223 |
| 5 | rs13478508 |  | 17 | rs4231504 |
| 5 | rs13478516 |  | 17 | UT_17_33.238924 |
| 5 | rs13478521 |  | 17 | rs13482974 |
| 5 | rs8262199 |  | 17 | rs4231504 |
| 5 | rs6319445 |  | 17 | rs8273969 |
| 5 | rs6336175 |  | 17 | rs13483042 |
|  |  |  | 17 | rs16806580 |
| 6 | rs13472831 |  | 17 | rs4231637 |
| 6 | rs3655269 |  | 17 | rs13483139 |
| 6 | rs13478656 |  | 17 | rs3687741 |
| 6 | rs3678887 |  | 17 | aEL-17_91401354 |
| 6 | rs16788669 |  | 17 | rs8252725 |
| 6 | rs29973570 |  | 17 | rs3696168 |
| 6 | mhcCD8b4 |  |  |  |
| 6 | rs3672029 |  | 18 | rs3656185 |
| 6 | rs16805672 |  | 18 | rs3691362 |
| 6 | rs13478891 |  | 18 | UNC_18_37970902 |
| 6 | rs16819438 |  | 18 | rs13483319 |
| 6 | rs3655148 |  | 18 | rs6364818 |
| 6 | rs4226162 |  | 18 | rs6350869 |
| 6 | rs16784854 |  | 18 | rs13483415 |
| 6 | rs6204829 |  | 18 | rs4231968 |
| 6 | rs13478997 |  |  |  |
| 6 | Snf06.122.747 |  | 19 | rs3671671 |
| 6 | rs8268650 |  | 19 | rs3713033 |
| 6 | rs6328711 |  | 19 | rs13483505 |
|  |  |  | 19 | rs6163293 |
| 7 | rs32212349 |  | 19 | rs3700209 |
| 7 | rs3689218 |  | 19 | rs6296521 |
| 7 | rs8276476 |  | 19 | rs6237846 |
| 7 | rs13479163 |  | 19 | CEL-19_12911424 |
| 7 | rs3719256 |  | 19 | rs3692733 |
| 7 | rs13479234 |  | 19 | rs3694570 |
| 7 | rs13479238 |  | 19 | UT_19_10.709331 |
| 7 | gnf07.050.858 |  | 19 | rs3669192 |
| 7 | rs3693038 |  | 19 | rs13483555 |
| 7 | rs8260829 |  | 19 | rs3720318 |
| 7 | rs13479274 |  | 19 | rs13483557 |
| 7 | rs13479276 |  | 19 | rs13459156 |
| 7 | rs13479277 |  | 19 | rs13483563 |
| 7 | rs13479317 |  | 19 | rs3653630 |
| 7 | Snf07.064.092 |  | 19 | rs13483571 |
| 7 | rs13479319 |  | 19 | rs13483577 |
| 7 | rs13479321 |  | 19 | rs13483600 |
| 7 | rs16808727 |  | 19 | rs4232223 |
| 7 | mSV25303361 |  | 19 | rs6257938 |
| 7 | rs3716002 |  | 19 | rs16791065 |
| 7 | rs16814851 |  |  |  |
| 7 | rs4226793 |  | X | rs13483723 |
| 7 | rs13479422 |  | X | rs6301011 |
| 7 | rs13479425 |  | X | CEL-X_44311522 |
| 7 | rs13479427 |  | X | rs13483753 |
| 7 | rs3691303 |  | X | rs13483757 |
| 7 | rs13479513 |  | X | rs16814469 |
| 7 | rs3687061 |  | X | rs13483831 |
| 7 | rs16807506 |  | X | CEL-X_68179178 |
| 7 | Snf07.129.013 |  | X | rs13483838 |
| 7 | rs3663988 |  | X | CEL-X_74985293 |
| 7 | rs8262638 |  | X | CEL-X_72697823 |
|  |  |  | X | CEL-X_74272691 |
| 8 | rs3701395 |  | X | rs6183945 |
| 8 | rs13479663 |  | X | CEL-X_91222960 |
| 8 | rs13479769 |  | X | rs13483935 |
| 8 | rs16822108 |  | X | rs13483941 |
| 8 | rs8236770 |  | X | rs13483969 |
| 8 | rs8254491 |  | X | rs6221690 |
| 8 | rs13479880 |  | X | rs13484004 |
| 8 | rs13479888 |  | X | rs13484050 |
| 8 | rs16793589 |  | X | rY6365259 |
| 8 | rs4227398 |  | X | rs8242429 |
| 8 | NCBI_08_131619152 |  | X | CEL-X_158112484 |
|  |  |  | X | NCBI_20_147612431 |
